# Automated diagnosis and classification of metacarpal and phalangeal fractures using a convolutional neural network: a retrospective data analysis study

**DOI:** 10.2340/17453674.2024.42702

**Published:** 2025-01-09

**Authors:** Michael AXENHUS, Anna WALLIN, Jonas HAVELA, Sara SEVERIN, Ablikim KARAHAN, Max GORDON, Martin MAGNÉLI

**Affiliations:** 1 Department of Orthopaedic Surgery, Danderyd Hospital, Stockholm; 2 Department of Clinical Sciences at Danderyd Hospital, Karolinska Institutet, Stockholm, Sweden

## Abstract

**Background and purpose:**

Hand fractures are commonly presented in emergency departments, yet diagnostic errors persist, leading to potential complications. The use of artificial intelligence (AI) in fracture detection has shown promise, but research focusing on hand metacarpal and phalangeal fractures remains limited. We aimed to train and evaluate a convolutional neural network (CNN) model to diagnose metacarpal and phalangeal fractures using plain radiographs according to the AO/OTA classification system and custom classifiers.

**Methods:**

A retrospective analysis of 7,515 examinations comprising 27,965 images was conducted, with datasets divided into training, validation, and test datasets. A CNN architecture was based on ResNet and implemented using PyTorch, with the integration of data augmentation techniques.

**Results:**

The CNN model achieved a mean weighted AUC of 0.84 for hand fractures, with 86% sensitivity and 76% specificity. The model performed best in diagnosing transverse metacarpal fractures, AUC = 0.91, 100% sensitivity, 87% specificity, and tuft phalangeal fractures, AUC = 0.97, 100% sensitivity, 96% specificity. Performance was lower for complex patterns like oblique phalangeal fractures, AUC = 0.76.

**Conclusion:**

Our study demonstrated that a CNN model can effectively diagnose and classify metacarpal and phalangeal fractures using plain radiographs, achieving a mean weighted AUC of 0.84. 7 categories were deemed as acceptable, 9 categories as excellent, and 3 categories as outstanding. Our findings indicate that a CNN model may be used in the classification of hand fractures.

With an aging population, the prevalence of hand fracture, including metacarpal and phalangeal fractures, is projected to increase in the coming years. The global incidence of hand fractures is substantial at 99 per 100,000 persons/year. Patients presenting with metacarpal and phalangeal fractures are a frequent occurrence in emergency departments (EDs) and misinterpretations of fractures can represent up to 24% of diagnostic errors in the ED [1].

A shortage of radiologists and an increased workload for radiological services has been observed in several countries [2]. Compared with the increase in ED visits, the increase in radiologists’ workload has more than doubled, sometimes growing up to tenfold, in several countries including Israel, Korea, the USA and the Netherlands [3]. Possible explanations include a more stressful work environment, leaving less time to conduct thorough clinical examinations, and less compliance with evidence-based imagery guidelines. In addition, radiology expertise is increasingly requested in diverse areas including acute stroke treatment and cancer screening [4]. It has been demonstrated that AI assistance can shorten a physician’s reading time significantly [5,6]. The implementation of such a program in clinical practice would theoretically leave radiologists with more time to perform other tasks. Moreover, a well-trained deep-learning (DL) model has the potential to be useful when expert interpretation of radiographs is not possible, such as in rural settings and ED clinics [7].

The applications and limitations of artificial intelligence (AI) in fracture detection and classification have shown promising outcomes, primarily in fractures of the ankle, hip, and spine [8]. However, little attention has been directed toward AI’s potential in identifying metacarpal and phalangeal fractures specifically. The potential for AI-assisted detection of hand fracture is apparent. Our study group has previously demonstrated that a DL program could detect hand fractures with an accuracy of 83% [9]. Despite this advancement, literature regarding AI’s role in diagnosing metacarpal and phalangeal fractures is lacking. We hypothesize that convolutional neural network (CNN) models can be effectively employed for diagnosing hand metacarpal and phalangeal fractures.

The aim of our study was to train and evaluate a CNN model to diagnose hand fractures using plain radiographs according to the AO/OTA classification system.

## Methods

### Study design and sample

In this retrospective study, 7,515 examinations comprising 27,965 images of metacarpal or phalangeal bones in a population aged 15 years or older were obtained from the Picture Archiving and Communication System (PACS) (Philips IntelliSpace PACS 4.4; Philips Healthcare, Best, Netherlands) at Danderyd University Hospital. The images were all obtained between 2002 and 2016 for clinical purposes. Each examination contained 2–9 radiographs. All images obtained were included, such as images with casts and implant Table 1 (see Appendix). Images used in this study varied in size and quality, reflecting the diversity of real-world clinical data. This variability was intentionally preserved to ensure the model’s robustness in handling typical clinical scenarios. All patient data was removed during the retrieval process. The study was reported according to the Transparent Reporting of a multivariable Prediction model for Individual Prognosis Or Diagnosis (TRIPOD) [10].

**Table 1:** Distribution of implants. Values are count and the percentage within that category in parentheses

Implant	Test (n = 327)		Validation (n = 415)		Training (n = 7,100)	
	Yes	No	Yes	No	Yes	No
All	12 (3.7)	315	2 (0.5)	413	110 (1.5)	6,990
Anchor					1 (0.0)	5,410
Cerclage					6 (0.1)	7,094
Hardware fracture					1 (0.0)	5,410
K-wires	2 (0.6)	325	1 (0.2)	414	25 (0.4)	7,075
Plate	5 (1.5)	322	1 (0.2)	414	72 (1.0)	7,028
Screws	4 (1.2)	323		415	29 (0.4)	7,071
Ex-fix	1 (0.3)	326			5 (0.1)	7,095
Failure to retrieve					1 (0.0)	5,410

### Datasets

The examinations were distributed between training (n = 7,100), validation (n = 415), and test (n = 327) datasets. There was no patient overlap between the datasets. The distribution of desired pathologies was aimed to be proportional across the training and validation sets. For the training dataset, examinations were initially randomly selected from the PACS. Thereafter, radiologists’ reports were searched for keywords in order to find examinations with hand fractures in categories with low prevalence in the training set. After intermittent testing against the validation dataset, further keyword searches were done to find examinations in categories with poor model performance. For the validation dataset, examinations were selected through keyword searches to find examinations with either hand fractures or normal findings. Retrieval and labelling of new examinations for the training dataset continued until intermittent testing ceased to reveal increased model performance. For the validation dataset, the process continued until fracture categories had an adequate number of cases for statistical analyses. The same patient could appear multiple times when examinations were performed 90 days apart, but there was no overlap between the training and validation sets. The radiographs used were anonymized and did not contain any patient data.

### Outcome variables

Classification of hand fractures was done according to AO/OTA and custom classifiers [11]. Images were labelled with anatomical location. There were 2 primary classification tasks: (1) binary classification, where the model distinguished between “fracture” and “no fracture” cases, and (2) multiclass classification, where the model further categorized the detected fractures into specific types according to the AO/OTA classification system and custom classifiers. Sensitivity and specificity were obtained for each categorized fracture localization.

### Review and labelling process

The retrieved examinations were uploaded to an in-house developed labelling platform where tools to label plain radiographic images were available. Original radiologists’ reports were included in most cases. 2 experienced senior orthopedic surgeons, blinded to the network’s predictions and initially independent of each other, classified the test set. Images with differing classifications were revisited, and consensus was reached after mutual discussion. 2 x 4th-year medical students from Karolinska Institutet, Stockholm, Sweden, labelled the training and validation sets under the supervision of a senior orthopedic surgeon. The students were trained by 2 senior orthopedic surgeons in classifying hand fractures and setting the labels. The students soon became independent in classifying, and they had access to the surgeon’s expert opinion if needed. Validation and consensus sessions were performed on parts of the training set and the validation set. The labels served as a ground truth. As network training was initiated, the network gradually learned to predict labels for each examination during subsequent labeling. The network’s predictions were incorporated into the online labeling platform and were presented to the human observers as a degree of network certainty ranging between < 50% certainty, 50–70% certainty, or > 70% certainty of selected labels. The human observers had the choice of keeping the network-predicted labels for the particular study or changing the labels based on their own assessment of the study.

### Fracture labeling and definitions

Fractures were categorized based on the AO/OTA classification system and custom classifiers (Table 2). Radiographs with multiple fractures were labeled accordingly using a hierarchical tree structure. Each fracture was classified into a type, and, where applicable, further categorized into a group, subgroup, and second subgroup. For example, fractures at the base of the fifth metacarpal were categorized into 5 distinct classes (a–e) (Table 3). Specifically, category “a” included all base fractures at metacarpal 5, category “b” encompassed all comminuted base fractures, category “c” included intra-articular comminuted fractures at the base of metacarpal 5, category “d” included simple base fracture at metacarpal 5, and category “e” included simple base fracture of metacarpal 5 of the Reverse Bennet type. Following this structured approach, we classified fractures across the hand into over 200 categories. The metacarpal bones were the most frequently fractured anatomical location, while fractures of the ulna were the least common (Table 4).

**Table 2:** Custom classifiers of hand fractures that are not described in the AO/OTA classification

Fracture	Description
Tuft	Fracture at the distal phalange
Bennett	Partial articular fracture at the base of metacarpal 1
Rolando	Complete articular fracture at the base of metacarpal 1
Reversed Bennett	Partial articular fracture at the base of metacarpal 5

**Table 3:** Example of fracture classification using our custom modifiers

Category	Type	Group	Subgroup	Second subgroup
a	Metacarpal 5	Base		
b	Metacarpal 5	Base	Comminute	
c	Metacarpal 5	Base	Comminute	Intra-articular
d	Metacarpal 5	Base	Simple	
e	Metacarpal 5	Base	Simple	Reverse Bennett

**Table 4:** Distribution of fractures, presence of dislocation, age of fracture, and presence of cast within the different datasets. Values are count and percentage in parenthesis within that category

	Test (n = 327)			Validation (n = 415)		Training (n = 7,100)		
	Yes	Maybe	No	Yes	No	Yes	Maybe	No
Fracture								
All	166 (51)	2 (0.6)	159 (49)	189 (46)	226 (55)	2,382 (34)	20 (0.3)	4,698 (66)
Bone								
Carpus	15 (4.6)		312 (95)	27 (6.5)	388 (94)	354 (5.0)	10 (0.1)	6,736 (95)
Metacarpal	91 (28)		236 (72)	80 (19)	35 (81)	733 (10)	2 (0.0)	6,365 (90)
Phalanx	50 (15)	2 (0.6)	275 (84)	52 (13)	363 (88)	616 (8.7)	4 (0.1)	6,480 (91)
Radius	22 (6.7)		305 (93)	36 (8.7)	379 (91)	710 (10)	4 (0.1)	6,386 (90)
Ulna	10 (3.1)		317 (97)	12 (2.9)	403 (97)	219 (3.1)		6,881 (97)
Location								
Base	28 (8.6)		299 (91)	17 (4.1)	398 (96)	212 (3.0)	1 (0.0)	6,887 (97)
Head/tuft	6 (1.8)		321 (98)	13 (3.1)	402 (97)	166 (2.3)	3 (0.0)	6,931 (98)
Intra-articular	28 (8.6)	2 (0.6)	297 (91)	28 (6.7)	387 (93)	396 (5.6)	3 (0.0)	6,701 (94)
Pattern								
Oblique	13 (4.0)		314 (96)	21 (5.1)	394 (95)	182 (2.6)	1 (0.0)	6,917 (97)
Shaft/waist	75 (23)		252 (77)	76 (18)	339 (82)	564 (7.9)	4 (0.1)	6,532 (92)
Spiral	24 (7.3)		303 (93)	12 (2.9)	403 (97)	69 (1.0)		7,031 (99)
Transverse	8 (2.4)		319 (98)	11 (2.7)	404 (97)	78 (1.1)		7,022 (99)
Cast								
All	57 (17)		270 (83)	57 (14)	358 (86)	798 (11)		6,302 (89)
Minimal	5 (1.5)		322 (96)	14 (3.4)	401 (97)	249 (3.5)		6,851 (97)
Plaster	48 (15)		279 (85)	43 (10)	372 (90)	549 (7.7)		6,551 (92)
Synthetic	5 (1.5)		322 (99)			6 (0.1)		7,094 (100)
Dislocation								
Fracture				3 (0.7)	412 (99)	23 (0.3)		7,077 (100)
Full	6 (1.8)		321 (98)	10 (2.4)	405 (98)	126 (1.8)		6,974 (98)
PIP	2 (0.6)		325 (99)	1 (0.5)	196 (99)	41 (0.8)		5,370 (99)
SL	3 (0.9)		324 (99)	2 (0.5)	413 (99)	32 (0.5)		7,068 (100)
Age of fracture								
All	25 (7.6)		302 (92)	11 (2.7)	404 (97)	222 (3.1)		6,878 (97)
1–2 weeks	2 (0.6)		325 (99)			33 (0.5)		7,067 (100)
Healed	18 (5.5)		309 (95)	7 (1.7)	408 (98)	108 (1.5)		6,992 (99)
Pseudarthrosis	7 (2.1)		320 (98)	4 (1.0)	411 (99)	97 (1.4)		7,003 (99)

PIP = proximal interphalangeal joint; SL = scapholunate joint.

### Model architecture and model training

We utilized the open-source machine learning framework PyTorch (v 1.13; https://pytorch.org/). We used a CNN of ResNet architecture consisting of 39 convolutional layers, each layer equipped with batch normalization, and an adaptive max pool as described by He et al. [12]. The architecture was chosen due to its effectiveness in image classification tasks, particularly in the domain of medical imaging. The ResNet model is known for its ability to handle deep networks by incorporating residual connections, which mitigate the vanishing gradient problem. This feature was critical for our study, which required a model capable of capturing fine-grained details across multiple fracture categories. The network was randomly initiated and trained using stochastic gradient descent. The training dataset labels served as ground truth during training, and examinations were processed by the network for 80 epochs. To increase the robustness of the training, each image was also rotated, flipped, and randomly cropped. The final CNN setup was used for testing the model against validation dataset labels (Table 5) [10].

**Table 5:** Neural network setup

Type	Blocks	Kernel size	Filters	Group	Layers
ResNet block	1	3x3	64	Image	2
ResNet block	1	3x3	64	Image	2
ResNet block	2	3x3	64	Core	4
ResNet block	2	3x3	128	Core	4
ResNet block	6	3x3	256	Core	12
ResNet block	6	3x3	512	Core	12
Average for top					
50% views	1	–	–	Pool	0
Convolutional	1	1x1	72	Classification	1
Fully connected	1	1x1	4	Classification	1
Fully connected	1	1x1	4	Classification	1

### Statistics

Statistical analysis was conducted using R software (v 4.3.0; R Foundation for Statistical Computing, Vienna, Austria). Model performance was evaluated through sensitivity, specificity, Youden’s index (J), and the area under the receiver operating characteristics (ROC) Curve (AUC) [13]. A 95% confidence interval (CI) was computed for the AUC value. Following the approach outlined by Mandrekar, an AUC of 0.5 indicates performance no better than chance, while 0.7–0.8 is considered acceptable, 0.8–0.9 excellent, and values exceeding 0.9 are deemed outstanding [14]. The ROC curve, depicting sensitivity on the Y-axis and 1-specificity on the X-axis, serves as a tool for assessing diagnostic test performance across various thresholds.

### Ethics, data sharing, funding, and disclosures

This study was approved by the Swedish Ethical Review Authority (2014/453-31). The raw datasets are available from the corresponding author on reasonable request.

No funding was received for this study. The study was financed by grants from the Swedish state under the agreement between the Swedish government and the county councils, the ALF-agreement, and student supervision reimbursement from Karolinska Institutet. The authors declare that they have no competing interests. Complete disclosure of interest forms according to ICMJE are available on the article page, doi: 10.2340/17453674.2024.42702

## Results

### Radiographs

7,515 examinations comprising 27,965 images were obtained. The examinations were distributed between training (n = 7,100), validation (n = 415), and test (n = 327) datasets.

Metacarpal fractures were the most prevalent in the test set of 327 examinations, making up 28% of cases. Phalangeal fractures were observed in 15% of cases, while carpal fractures accounted for 4.6%. Radius fractures were present in 6.7% of cases, and ulna fractures were the least common, occurring in 3.1% of cases. In the validation set of 415 examinations metacarpal fractures were the most common, accounting for 19% of the cases, followed by phalangeal fractures at 13%. Carpal fractures were observed in 6.5% of cases, and radius fractures were present in 8.7%. Ulna fractures were the least common, occurring in 2.9% of cases. Metacarpal fractures were also the most frequent in the training set of 7,100 examinations, accounting for 10% of cases. Phalangeal fractures were observed in 8.7% of cases, while carpal fractures made up 5.0%. Radius fractures were present in 10% of the cases, and ulna fractures were relatively uncommon, occurring in 3.1% of cases (Figure, Table 4).

**Figure:**
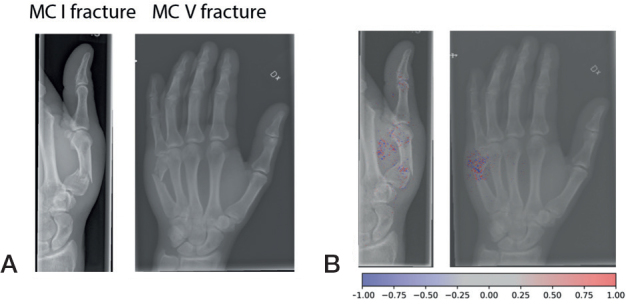
Examples of fractures grades correctly graded by the network (A). Network gradient is provided (B).

### The CNN model

The model demonstrated varying levels of diagnostic accuracy across different fracture categories. 3 categories were deemed to be outstanding, 9 categories excellent, and 7 categories acceptable. While the overall fracture detection yielded an AUC of 0.79, with a sensitivity of 80% and specificity of 63%, performance varied significantly across specific fracture types. Transverse fractures of the metacarpal bones showed an AUC of 0.91, with 100% sensitivity and 87% specificity, and tuft phalangeal fractures demonstrated an AUC of 0.97, with 100% sensitivity and 96% specificity. However, certain categories, such as oblique fractures of the phalanges, had a lower performance (AUC of 0.76, with 71% sensitivity and 79% specificity). We detected only 15 carpal bone fractures with 13 scaphoid and 2 triquetrum fractures (Table 6). Mean weighted summary statistics showed an AUC of 0.84 for hand fractures (Table 7).

**Table 6:** Network performance in diagnosing and classifying finger fractures

Item	Cases (n = 327)	Sensitivity (%)	Specificity (%)	Youden’s J	AUC (CI)	Accuracy (%)
All	168	80	63	0.43	0.79 (0.74–0.84)	71
Metacarpal						
All	91	77	91	0.68	0.89 (0.85–0.93)	87
Base	28	96	43	0.40	0.74 (0.66–0.83)	50
Comminuted	17	65	93	0.57	0.84 (0.75–0.94)	91
Oblique	6	100	76	0.76	0.90 (0.83–0.97)	76
Simple	48	81	86	0.67	0.88 (0.83–0.93)	85
Spiral	24	92	67	0.58	0.81 (0.73–0.88)	68
Transverse	7	100	87	0.87	0.91 (0.88–0.95)	87
Shaft	48	83	85	0.69	0.88 (0.83–0.93)	85
Neck	17	88	78	0.66	0.86 (0.79–0.94)	78
Intra-articular	7	100	67	0.67	0.82 (0.72–0.91)	67
Phalanges						
All	52	92	62	0.54	0.81 (0.75–0.87)	71
Base	33	74	69	0.43	0.74 (0.66–0.82)	69
Close to PIP	18	76	68	0.45	0.74 (0.61–0.87)	69
Oblique	7	71	79	0.50	0.76 (0.59–0.93)	79
Tuft	3	100	96	0.96	0.97 (0.95–1.00)	96
Shaft	17	76	84	0.60	0.84 (0.74–0.94)	83
Intra-articular	23	95	44	0.40	0.72 (0.62–0.82)	47
Carpal						
Scaphoid	13	77	91	0.68	0.90 (0.82–0.98)	90
Triquetrum	2	100	65	0.65	0.82 (0.48–1.00)	65
Fracture type						
Base	28	93	46	0.39	0.74 (0.66–0.83)	50
Distal	18	89	80	0.69	0.91 (0.84–0.97)	80
Head/tuft	6	83	86	0.69	0.80 (0.54–1.00)	86
Intermediate	7	50	92	0.42	0.67 (0.39–0.96)	91
Oblique	13	85	58	0.43	0.75 (0.64–0.86)	59
Proximal	29	79	68	0.46	0.76 (0.68–0.85)	68
Proximal base extra-articular	7	71	79	0.51	0.77 (0.59–0.94)	79
Shaft neck/shaft	12	83	91	0.74	0.85 (0.71–0.98)	90
Shaft/waist	75	83	70	0.53	0.81 (0.76–0.86)	72
Spiral	24	92	64	0.56	0.80 (0.72–0.88)	66
Transverse	8	100	77	0.77	0.90 (0.84–0.95)	77
Shaft	5	100	90	0.90	0.96 (0.92–1.00)	76
Intra-articular	30	71	69	0.41	0.72 (0.64–0.81)	69

**Table 7:** Mean weighted summary statistics. Each analysis is multiplied by the number of cases and then divided by the total case number

Statistics	Mean
Accuracy	0.77
Sensitivity	0.86
Specificity	0.76
Youden J	0.62
AUC	0.84

## Discussion

The aim of this study was to explore the potential of a CNN model in diagnosing and categorizing mainly metacarpal and phalangeal fractures according to the AO/OTA classification system and by custom classifiers from plain radiographs. While carpal fractures were present in the dataset and analyzed for completeness, the primary aim was to evaluate the model’s performance in diagnosing and categorizing metacarpal and phalangeal fractures. Our findings showed that 7 categories were deemed to be acceptable, 9 categories excellent, and 3 categories outstanding. In addition, we further established that the program varies in its capability to classify specific fractures, depending on which bone is affected.

Fracture classification using AI has been shown to be effective and accurate. In a previous study, we showed that a similar AI model can obtain excellent results in shoulder fracture classification [15]. In a meta-analysis on fracture detection using AI, Kuo et al. observed a pooled sensitivity of 91% and specificity of 91% [16]. In a review by Langerhuizen et al., the authors noted AUC ranging from 77–90% depending on study design and fracture type [8]. However, these studies focused on body parts other than hands, and the only programs that classified fractures did so on diaphyseal femur fractures.

Current research on AI and the detection and classification of metacarpal and phalangeal fractures is scarce. When comparing our results with those of Üreten e. al., who trained a DL program to diagnose phalangeal fractures, we have similar accuracy (Üreten 84% vs our AUC of 0.81), better sensitivity (84.1% vs 90%), and inferior specificity (83.3% vs 62%) [17]. Meanwhile, Olczak et al. managed to diagnose the presence of a hand fracture with an accuracy of 83% [9]. Our results for carpal fractures are similar to those of Ozkaya et al., with a sensitivity of 77% (vs 76%), specificity of 79% (vs 92%), AUC of 0.82 (vs 0.84), and J of 0.56 (vs 0.68) [18]. Ozkaya et al. is the only published study to our knowledge that also developed a DL program which could both identify and classify carpal fractures and thus can be considered the most comparable study available. Our results are therefore in line with existing research on AI identification of phalangeal fractures with a more defined classification of fractures although with a lower detection rate. It is likely that our lower detection rate is due to our varied dataset, which included suboptimal examinations, such as images with casts, in order to improve robustness. The observed high specificity in categories such as metacarpal transverse fractures and tuft phalangeal fractures reflects our model’s proficiency in detecting clearly defined, less ambiguous fracture patterns. However, the model’s performance was lower in more complex fracture types, such as oblique and intra-articular fractures, where the specificity and sensitivity were not as high. These findings underscore the importance of considering the specific characteristics of fracture types when evaluating the utility of AI models in clinical practice. Future iterations of the model could benefit from targeted training on more challenging fracture types and external validation to improve overall diagnostic accuracy.

### Strengths

One of the strengths of our approach lies in the extensive inclusion of examinations. In contrast to previous studies with fewer examinations, our study incorporated a larger dataset, enhancing its robustness. Additionally, the diversity of projections in each examination, reflective of real clinical scenarios, distinguishes our study from others employing standardized databases.

### Limitations

Two 4th-year medical students were responsible for categorizing the examinations. This may have resulted in inaccuracy and skewness of the model’s effectiveness and practicality. To address this concern, initial labeling was conducted under the guidance of an orthopedic surgeon, with challenging cases revisited under supervision to ensure accuracy. Furthermore, external validation of our model is required in order to enhance its generalizability.

### Conclusion

We found that it is possible to train and evaluate a convolutional neural network (CNN) model in order to diagnose hand fractures using plain radiographs. We showed that 7 categories were deemed to be acceptable, 9 categories excellent, and 3 categories outstanding. In addition, we further established that the program varies in its capability to classify specific fractures depending on which bone is affected.

*In perspective,* our findings indicate that a CNN model may be used in the classification of hand fractures.

## Notes

Conceptualization: MG, MM. Methodology: MG, MM. Software: MA, MG, MM. Validation: MG, MM. Formal analysis: MA, JH, AW, MM. Investigation: JH, AW, MM, AK. Resources: MA, MG, MM. Data curation: MG, MM. Writing original draft: MA. Writing—review & editing: MG, MM, AK, SS, AW, JH. Visualization: MA, JH, MM. Supervision: MA, MG, MM.

Handling co-editors: Taco Gosens

*Acta* thanks anonymous reviewers for help with peer review of this manuscript.
